# Polypharmacy and Quality of Life Among Dialysis Patients: A Qualitative Study

**DOI:** 10.1016/j.xkme.2023.100749

**Published:** 2023-11-28

**Authors:** Julia M.T. Colombijn, Freek Colombijn, Lideweij van Berkom, Lia A. van Dijk, Dionne Senders, Charlotte Tierolf, Alferso C. Abrahams, Brigit C. van Jaarsveld

**Affiliations:** 1 Department of Nephrology and Hypertension, University Medical Center Utrecht, Utrecht, the Netherlands; 2 Julius Center for Health Sciences and Primary Care, University Medical Center Utrecht, Utrecht University, Utrecht, the Netherlands; 3 Department of Nephrology, Amsterdam UMC, Vrije Universiteit Amsterdam, Research Institute Amsterdam Cardiovascular Sciences, Amsterdam, the Netherlands; 4 Department of Social and Cultural Anthropology, Vrije Universiteit Amsterdam, Amsterdam, the Netherlands; 5 Diapriva Dialysis Center, Amsterdam, the Netherlands

**Keywords:** Dialysis, medication, patient experiences, polypharmacy, qualitative research

## Abstract

**Rationale & Objective:**

Almost all patients who receive dialysis experience polypharmacy, but little is known about their experiences with medication or perceptions toward it. In this qualitative study, we aimed to gain insight into dialysis patients’ experiences with polypharmacy, the ways they integrate their medication into their daily lives, and the ways it affects their quality of life.

**Study Design:**

Qualitative study using semistructured interviews.

**Setting & Participants:**

Patients who received dialysis from 2 Dutch university hospitals.

**Analytical Approach:**

Interviews were transcribed verbatim and analyzed independently by 2 researchers through thematic content analysis.

**Results:**

Overall, 28 individuals were interviewed (29% women, mean age 63 ± 16 years, median dialysis vintage 25.5 [interquartile range, 15-48] months, mean daily number of medications 10 ± 3). Important themes were as follows: (1) their own definition of what constitutes “medication,” (2) their perception of medication, (3) medication routines and their impact on daily (quality of) life, and (4) interactions with health care professionals and others regarding medication. Participants generally perceived medication as burdensome but less so than dialysis. Medication was accepted as an essential precondition for their health, although participants did not always notice these health benefits directly. Medication routines and other coping mechanisms helped participants reduce the perceived negative effects of medication. In fact, medication increased freedom for some participants. Participants generally had constructive relationships with their physicians when discussing their medication.

**Limitations:**

Results are context dependent and might therefore not apply directly to other contexts.

**Conclusions:**

Polypharmacy negatively affected dialysis patients’ quality of life, but these effects were overshadowed by the burden of dialysis. The patients’ realization that medication is important to their health and effective coping strategies mitigated the negative impact of polypharmacy on their quality of life. Physicians and patients should work together continuously to evaluate the impact of treatments on health and other aspects of patients’ daily lives.

**Plain-Language Summary:**

People receiving dialysis treatment are prescribed a large number of medications (polypharmacy). Polypharmacy is associated with a number of issues, including a lower health-related quality of life. In this study we interviewed patients who received dialysis treatment to understand how they experience polypharmacy in the context of their daily lives. Participants generally perceived medication as burdensome but less so than dialysis and accepted medication as an essential precondition for their health. Medication routines and other coping mechanisms helped participants mitigate the perceived negative effects of medication. In fact, medication led to increased freedom for some participants. Participants had generally constructive relationships with their physicians when discussing their medication but felt that physicians sometimes do not understand them.

People receiving dialysis treatment (hereafter dialysis patients) are prescribed a large number of medications (ie, polypharmacy) to manage comorbid conditions and improve metabolic control.^1^ Polypharmacy is associated with a high prevalence of non-adherence to medication and the development of medication-related problems, such as medication errors, adverse drug events, and drug-drug interactions.^2^^,^^3^

Results from quantitative studies, including one of our studies, suggest that polypharmacy is also associated with lower health-related quality of life (HRQoL) in dialysis patients.^4^^,^^5^ These studies rely on general quality of life questionnaires that are well-suited to compare the HRQoL scores of different study populations and to study associations between exposures and HRQoL. However, these studies are less useful in providing insight into patients’ personal experiences with polypharmacy and the way patients give meaning to their medication use. Qualitative studies can fill this void and analyze the impact of medication in the context of patients’ daily lives.^6^

Two systematic reviews of qualitative studies on patients’ experiences with polypharmacy report that polypharmacy causes various practical issues for patients and can also negatively affect their self-perception and daily lives.^7^^,^^8^ However, neither review included studies conducted with dialysis patients. Experiences from other patient populations with polypharmacy might not be generalizable because dialysis patients usually have more extreme levels of polypharmacy than other patient populations; the dialysis treatment complicates medication regimens because patients have to take medication at set times and consider the clearance of medication through dialysis.^9^ Most importantly, dialysis is by itself a very burdensome treatment that significantly affects HRQoL.^10^

Therefore, this follow-up study aimed to fill important gaps in both quantitative and qualitative studies to gain insight into dialysis patients’ experiences with polypharmacy. It will deepen our understanding of dialysis patients’ perceptions of their medication, the ways they integrate medication into their daily lives, and how it affects their quality of life.

## Methods

### Study Design

This qualitative study was embedded in the Dutch nOcturnal and hoME dialysis Study To Improve Clinical Outcomes (DOMESTICO).^11^ DOMESTICO is a prospective cohort study in Dutch and Belgian dialysis centers that compares the HRQoL and clinical outcomes of incident patients treated with home and in-center dialysis. This study was reported according to the consolidated criteria for reporting qualitative research (COREQ).^12^ The protocol was approved by the Amsterdam University Medical Centre Medical Ethics Review Board (approval number 2021.0113). All participants provided informed consent in writing.

### Participants

Patients were eligible to participate in the present study if they were aged ≥18 years and had been receiving dialysis for at least 3 months. Patients insufficiently proficient in Dutch or English were excluded. Patients from the dialysis clinics of the Amsterdam University Medical Centers the University Medical Center Utrecht and the affiliated dialysis centers Diapriva and Dianet were sampled. Patients were approached to participate by their nephrologist. We purposely approached patients who were being treated with different dialysis modalities (day- or night-time in-center hemodialysis and peritoneal dialysis) to obtain a more diverse study population. The response rate was high with only 2 patients declining to participate: one thought the interviews were too time-consuming and another felt uncomfortable participating. After the interview, participants received a gift voucher, but this had not been used as an incentive when they were invited to participate.

### Data Collection

JC, FC, LvB, LvD, DS, and CT conducted semistructured interviews in pairs from April to July 2021 based on an interview guide. Interviewing in pairs facilitates asking follow-up questions as interviewers can take turns. All interviewers have a background in cultural anthropology, except JC (medicine and health sciences). None of the interviewers had personal experience with dialysis or polypharmacy nor had anybody in their social circle. The interviewers were neither involved in the care of participants nor otherwise associated with them.

The interview guide was developed a priori and updated during the study if new themes emerged from the interviews. The team of interviewers held weekly meetings during the first weeks of data collection to calibrate the questions and possible directions for follow-up questions. Topics discussed included daily medication and dialysis routines, participants’ definition of “quality of life,” and experience with medication (for the full interview guide see Table S1). The names of the medications were used if the participants were familiar with them; otherwise, a description in colloquial language was used.

To minimize the inconvenience for participants and to stimulate rapport between researchers and participants, the latter chose the location of the interview. Patients were interviewed in the dialysis ward (n = 15), at home (n = 10), in a consulting room (n = 2), and a roadside restaurant (n = 1). Interviews were in Dutch and lasted approximately 1-2 hours. Seventeen participants were interviewed once and 11 twice. Interviews were audio-taped and transcribed verbatim unless participants objected (n = 4). Interviews without audio-tape were documented using notes made during the meeting. Interviews were supplemented by medical data from patients’ medical records, drawings of social networks patients made on invitation, observations made during the interview, and the published memoir of 1 patient. Data collection was stopped when data saturation was reached.

### Data Analysis

Descriptive analyses were performed in R studio (version 4.0.3). Two researchers (JC and FC) analyzed the transcripts independently using thematic content analysis. A coding tree was developed from themes emerging from the transcripts. The transcripts were then recoded according to the coding tree, and the final coding results were compared. Disagreements about themes, the coding tree, and final coding were resolved by discussion. Taking the interviews as a starting point for the coding instead of a preconceived framework allowed patterns and themes to emerge from the data bottom-up. Patients’ expressions quoted in this article were translated into English (see Table S2 for original quotes). In addition, to illustrate the coherence between themes, we present the life histories of 2 participants (“Michael” and “Catherine”).

## Results

### Participants

Twenty-eight patients participated in the study. Mean age was 63 ± 16 years; 20 identified as men (71%) and 8 as women (29%) (Table 1). Participants had a median dialysis vintage of 25.5 (interquartile range [IQR], 15-48) months; 19 received hemodialysis (68%) and 9 peritoneal dialysis (32%). The daily number of medications and pill burden were 10 ± 3 and 14 ± 6, respectively. Most participants took medication 3 or 4 times a day.

**Table 1:** Characteristics of Participants

	All Participants (n = 28)	Hemodialysis (n = 19)	Peritoneal Dialysis (n = 9)
**Demographics**			
Age (y)	63 ± 16	62 ± 16	64 ± 16
Gender			
*Male*	20 (71)	13 (68)	7 (78)
*Female*	8 (29)	6 (32)	2 (22)
*Nonbinary*	0 (0)	0 (0)	0 (0)
Household composition			
*Composite household*	18 (64)	13 (68)	5 (56)
*Singular household*	10 (36)	6 (32)	4 (44)
Employment status			
*Employed*	8 (29)	4 (21)	2 (22)
*Unemployed*	6 (21)	4 (21)	4 (44)
*Retired*	14 (50)	11 (58)	3 (34)
**Medical history**			
Dialysis modality			
*In-center daytime hemodialysis*	16 (57)	16 (84)	0 (0)
*In-center nocturnal hemodialysis*	3 (11)	3 (16)	0 (0)
*CAPD*	4 (14)	0 (0)	4 (44)
*APD*	5 (18)	0 (0)	5 (56)
Dialysis vintage (mo)	25.5 (15-48)	31 (17-53.5)	18 (15-26)
Primary kidney disease			
*Diabetes/renovascular kidney disease*	15 (54)	9 (47)	5 (56)
*Cystic kidney disease*	5 (18)	3 (16)	2 (22)
*Glomerulonephritis*	4 (14)	3 (16)	1 (11)
*Other/unknown*	4 (14)	4 (21)	1 (11)
History of diabetes mellitus	12 (43)	8 (42)	4 (44)
History of cardiovascular disease	10 (38)	5 (26)	4 (44)
**Medication history**			
Total number of medications^a^	14 ± 3	15 ± 3	13 ± 3
Daily number of medications	11 ± 3	10 ± 3	12 ± 3
Daily pill burden	14 ± 5	14 ± 6	15 ± 4
Daily number of intake moments			
*1*	3 (11)	3 (16)	0 (0)
*2*	5 (18)	3 (16)	2 (22)
*3*	16 (57)	10 (52)	6 (67)
*4*	4 (14)	3 (16)	1 (11)

*Note:* Continuous variables were described as mean ± standard deviation or median (IQR). Categorical variables were described as frequency (percentage).

Abbreviations: APD, automated peritoneal dialysis; CAPD, continuous ambulatory peritoneal dialysis; IQR, interquartile range.

a Medications include all prescribed medication, including those administered during dialysis.

Themes that emerged from the analysis were as following: (1) the definition of medication; (2) the perception of medication (subthemes: amount of medication, effects on health, and physical discomforts); (3) medication routines and its impact on daily life (subthemes: storage, taking medication, and adherence); and (4) interactions with health care professionals and others regarding medication. Illustrative quotes for these themes are presented in Table 2.

**Table 2:** Selected Quotes From Interviews Reflecting the Experiences of Patients Receiving Dialysis with Polypharmacy

Quote
**Perception of medication**
*“I feel so terribly dependent on it.”* (R25, male, 86 y, 12 medications, 12 pills)
*“I have always learnt: you have to find your own solution*.*”* (R6, male, 83 y, 11 medications, 8 pills)
*“I am part of nature, but I swallow chemical garbage.”* (R1, male, 55 y, 15 medications, 21 pills)
*“I would rather not take it [medication] … But I cannot live without it, so you can grumble all you like but that does not help.”* (R18, female, 76 y, 16 medications, 14 pills)
*“It’s part of daily life. I mean, there are also other people who have to take medication. It’s all part of the package*.*”* (R10, female, 61 y, 14 medications, 11 pills)
*“It is my life-saver! I am so grateful that I … still have this chance [in my life]. It is the medication…I’m for it.”* (R12, male, 71 y, 11 medications, 7 pills)
*“You have to take your medication because you would destroy yourself if you didn’t …then you’ve had it*.*”* (R15, male, 73 y, 17 medications, 18.5 pills)
*“For me it is like there is no other way. You must do this or you will have to put on a wooden coffin and your suffering will be over*. *But I’m not ready to do that*.*”* (R3, male, 79 y, 18 medications, 23 pills)
*“I just don’t like having to place those pills next to your plate everywhere [you go]. Maybe a sense of shame … a kind of embarrassment that I happen to be the patient, the feeble one, the vulnerable one.”* (R19, male, 53 y, 13 medications, 14 pills)
“*I am not going to stand up in the middle of a [restaurant] to take pills, but I am not ashamed of it either. [Taking pills in public] doesn’t bother me at all.”* (R24, male 67 y, 17 medications, 15.5 pills)
**Medication routines and the impact of medication on daily life**
*“You do not forget to eat, so, yes, this little basket is always on the table.”* (R27, female, 41 y, 14 medications, 14.5 pills)
*“Sunday is pill day.”* (R3, male, 79 y, 18 medications, 23 pills)
*“It is automatic… I don’t even think about it*.*”* (R16, male, 87 y, 16 medications, 21 pills)
*“Everything in one go, gulp of water, done*.*”* (R24, male, 67 y, 17 medications, 15.5 pills)
*“Ideally, I have nothing to do with dialysis during the day. During the evening and at night, I am willing to have it, but during the day I just want to be free without having to think ‘Oh, I have to take a little pill’*.*”* (R19, male, 53 y, 10 medications, 13 pills)
*“I take it [medication], and after that I forget I am ill.”* (R23, male, 59 y, 11 medications, 12 pills)
**Interaction with health care workers and social environment regarding medication**
*“That doctor looks at which medication is good for me… “Then that will be all right”, is what I say. Doctors know better than I do.”* (R29, male, 60 y, 19 medications, 23 pills)
*“The doctor ordered me to.”* (R2, female, 70 y, 16 medications, 10 pills)
*“From time to time, I think ‘swallow this yourself just once, so you know how it feels’ because sometimes it is so difficult to explain.”* (R1, male, 55 y, 15 medications, 21 pills)
*“I want to know what pills I am taking, and for what, and if they don’t work, I sound the alarm because I do not want to swallow anything I do not need to.”* (R18, female, 76 y, 16 medications, 14 pills)
*“I don’t think I am a guinea pig, just take medication and wait and see if it works.”* (R23, male, 59 y, 11 medications, 12 pills)

### Definition of Medication

Patients and nephrologists can have a different understanding of what they mean when they speak of “medication.” One participant, for example, described medication as “*serious business*,” and another declared that medication was specifically intended to cure disease. Following this logic, vitamin supplements, laxatives, phosphate binders, paracetamol, and creams were explicitly excluded from these definitions. There were also participants (such as Michael, Box 1) who contrariwise considered several products as medication that nephrologists do not typically consider medication, such as herbal preparations (eg, jungle leaves or herbal teas) and uncontaminated urine. Different definitions of medication can cause misunderstandings between patients and nephrologists when they discuss medication.

Box 1 **Portrait of Michael, a Dutch-Surinamese Man Who is Very Skeptical About his Nephrologists and Western Medicine.**

Michael is a Dutch-Surinamese man in his 40s who is divorced and lives on his own in a lower-middle-class neighborhood of Amsterdam. He has 3 children, but only has contact with his youngest child. He leads a quiet life. He spends his days, apart from going to the dialysis center 3 times a week, walking, doing household chores, and watching television. He is a devout Christian who cherishes his African and Surinamese roots. He has travelled to Africa multiple times and returns to Suriname every year where he feels the food and climate improve his health.

The first thing he said after we invited him to participate in this study was “*You’re probably not interested in interviewing me. My opinion about medication is very different to most patients*.” Michael believes that his kidney problems stem from slime plugs caused by a bad diet that block his kidneys. He managed to postpone dialysis for 8 years until 2012, when he experienced a life-threatening illness, by treating himself with medicinal leaves from Suriname, which he purchased there during visits or are sent to him by his family in Suriname. Since then, he has received hemodialysis.

Michael has an ambivalent attitude towards his nephrologists. In his view, God, not his nephrologists, presides over his health, saying “*I am religious. I believe in God… I thought, you [the nephrologist] are not God. Only a doctor.”* He is skeptical about his nephrologists’ medical competence. Nevertheless, he feels indebted to them for their efforts in keeping him alive, saying *“I thought, they [nephrologists] are just tinkering. They’re only human. They mess about a lot. I am glad they keep me alive and should be grateful for that. If it were not for the dialysis, I would be gone by now. But I don’t understand why they don’t look at the other side of the coin. Herbs and spirituality, etc*.*”* He has no confidence in his medication: “*Pills have not done me any good.… I have tried all kinds of pills, but they are just chemical garbage. As a patient, you are desperate. They prescribe you pills as if you were a guinea pig. It felt as if my medication made my body run haywire*.” Michael has embraced his lack of agency on his destiny and actively rejects treatment he considers futile: *“I am not going to follow it [the treatment]. I go [to the Hereafter] only once, and when I go, I shall go in peace. It is out of our hands*.” Instead, he devotedly adheres to his own “medication” of fruit, vegetables, leaves, and herbs. Overall, he feels that his body is slowly recovering thanks to his treatment.

Although patients and nephrologists can have different understandings of what constitutes “medication,” most participants in our research were well aware which medications (in the nephrologists’ definition) they were taking. Many knew the exact names, could give the reasons for prescription, and were familiar with additional intake instructions (such as to take during meals).

### Perception of medication

Most participants talked about the medication as a necessary evil. One person sighed, “*I feel so terribly dependent on it*.” (participant 25). Although almost all participants thought their medication was less burdensome than dialysis, most wished they could take less, and some actively argued they were “antipill” because “*I have always learnt: you have to find your own solution.*” (participant 6). Others, including Michael (Box 1), dismissed their medication as garbage or poison. As one participant said, “*I am part of nature but I swallow chemical garbage.*” (participant 1).

However, most had resigned themselves to taking medication for the rest of their lives. Resisting or complaining about medication would be futile because there was no prospect of a life without medication. “*I would rather not take it [medication]…. But I cannot live without it, so I could grumble about it but that does not help*.” (participant 18).

One participant took this a step farther as she insisted that taking medication was necessary to many people, not only dialysis patients: “*It’s part of daily life. I mean, there are also other people who have to take medication. It’s all part of the package.*” (participant 10). Another was more positive: “*It is my life-saver! I am so grateful that I … still have this chance [in my life]. It is the medication…I’m for it.*” (participant 12).

#### Amount of Medication

Most participants felt they had to take a lot of medication. Participants measured “a lot” relative to their expectations rather than to an absolute standard. Some mockingly called themselves “the pharmacy,” and several reported that they had to take so much medication that they would not notice the difference if they had to take a couple of extra pills. Participants with more (severe) medication regimens in the past had a more favorable outlook on their current amount of medication. However, respondents who were prescribed more medication over time did not necessarily hold more negative views. Their views evolved as they became accustomed to the large amount of medication.

#### Effects on Health

Participants’ perception of medication is influenced by the degree they feel the positive effects of their medication. Many participants expressed difficulty noticing the efficacy of medications or to distinguish the effects of dialysis. The effects of medication, such as analgesics or sleeping tablets, on symptom relief were easiest to perceive. Several participants also reported that they felt an increase in energy levels from their erythropoietin-stimulating agents. The effects of medications with more subtle effects, such as loop diuretics, were noticeable only when participants skipped a dose. However, participants did not notice the effects of most medications or only indirectly through improvements in blood pressure or laboratory measurements.

Despite generally negative perceptions of medication and a lack of directly noticeable positive effects, almost all participants emphasized that not taking their medication would lead to (further) deterioration in their health and even death. As one participant said, “*You have to take your medication because you would destroy yourself if you didn’t … then you’ve had it.*” (participant 15). Another mentioned, “*For me it is like there is no other way. You must do this or you will have to put on a wooden coffin, and your suffering will be over. But I’m not ready to do that.*” (participant 3).

Medication negatively affected the self-perception of some participants who reasoned how one could possibly be healthy if one had to take medication. In that sense, they interpreted medication as a symptom of physical failure, not a remedy. One participant felt ashamed to take medication visibly in front of others, stating “*I just don’t like having to place those pills next to your plate everywhere [you go]. Maybe a sense of shame … a kind of embarrassment that I happen to be the patient, the feeble one, the vulnerable one*” (participant 19). A self-confident participant, in contrast, took his pills openly when having dinner with his friends: “*I am not going to stand up in the middle of a [restaurant] to take pills, but I am not ashamed of it either. [Taking pills in public] doesn’t bother me at all.*” (participant 24).

#### Physical Discomforts

The general perception of medication as something unpleasant, albeit inescapable, was aggravated by physical discomforts. In some participants, medication intake triggered unpleasant physical reactions, such as retching and difficulty swallowing. One participant had to sit down for a while after taking her medication to give feelings of malaise time to subside, and another, conversely, felt the urge to move to divert attention away from the medication. Other participants were concerned about the fact that phosphate-binder tablets left a dry taste and that potassium resins made them feel like they were swallowing sand.

Physical discomforts were not limited to administering medication. Although several participants did not experience side effects, the majority reported a myriad of side effects ranging from dry, itchy skin to dizziness (hypotension and hypoglycemia), bruises, diarrhea, indefinable restlessness, feelings of depression or grumpiness, and loss of libido. For participants on a fluid restriction, medications that have to be taken with a lot of water (because the pills are difficult to swallow or because the medication has to be dissolved in water) were particularly problematic because they had to use up a significant part of their already limited fluid rations on their medication intake.

### Medication Routines and the Impact of Medication on Daily Life

Participants had different methods of organizing their medication and simplifying their medication intake.

#### Storage

The simplest routine patients followed was to keep the original medication boxes together in a shopping bag or a cupboard. One participant kept her medication in a basket on the dining table as a mnemonic tool: “*You do not forget to eat, so, yes, this little basket is always on the table.*” (participant 27).

More sophisticated methods were pill organizers that participants prepared themselves or Baxter rolls (with medication sachets for each moment patients have to take medication prepared by the pharmacy). These more sophisticated methods have 3 advantages: first, participants can easily verify if they have forgotten a dose; second, they can easily take their medication with them wherever they go; and third, preparing the medication in advance saves time. “*Sunday is pill day*”, one participant (participant 3) stated. One participant deliberately rejected Baxter rolls because the pharmacy was too slow adapting the rolls to changes in his prescriptions.

#### Taking Medication

Partly as a result of their careful preparation, participants spent little time on their medication during the day. Most participants reported no issues with taking the medication: “*It is automatic … I don’t even think about it.*” (participant 16). Some even took 10 pills simultaneously: “*Everything in one go, gulp of water, done*” (participant 24). Others found it difficult to swallow pills or used drinks or food to mask the unpalatable taste and chemical smell of medication.

Most medication routines focused on taking the medication at set times so participants did not have to think about their medication during the day. For the same reason, participants preferred combining medication intake with dialysis (particularly those patients who received home dialysis): “*Ideally, I have nothing to do with dialysis during the day. During the evening and at night, I am willing to have it, but during the day I just want to be free without having to think ‘Oh, I have to take a little pill’.*” (participant 19). Efficient medication routines allowed participants to take their minds off being ill for large parts of the day. One participant went as far as to say: “*I take it [medication], and after that I forget I am ill*” (participant 23). However, participants felt annoyed when medication disrupted their sense of a normal life, for instance, if they had to stay at home waiting for deliveries from the pharmacy.

Medication could also restore some other treasured routines. For instance, one participant used her potassium resins to allow her to eat more potassium-rich foods. Other participants treasured medication that improved their quality of sleep and consequently increased their energy levels, allowing them to participate in more activities during the day. Medication also helped to preserve bodily functions, such as diuresis, giving them the feeling of a normal life.

#### Adherence

Most participants said they took their medication as prescribed. With one exception, people who resented their medication also took their medication because they felt that they could not live without it. Medication routines helped participants to take the medication in time or to alert them if they had forgotten. Multiple participants were not bothered if they missed or skipped a dose. Thanks to their routines, they would quickly realize this and then take their medication.

### Interaction With Health care Professionals and Social Environment Regarding Medication

#### Health care Professionals

Approximately 3 patterns can be discerned in the interaction with health care professionals. These patterns are not strictly separated, and participants sometimes alternated between different patterns. Which pattern characterized the relationship between a particular patient and health care professional was influenced by the balance of power between them; this in turn was influenced by participants’ intellectual or social resources.

In the first pattern, a docile patient accepted the nephrologist’s authority and relied fully on their advice: “*That doctor looks at which medication is good for me … ‘Then that will be all right,’ is what I say. Doctors know better than I do*” (participant 29). These participants did not feel qualified to take decisions on medication and simply do things because “*the doctor ordered me to*” (participant 2). Patients following this pattern often do not bring up any problems they experience or that they feel helpless about communicating their emotions. Consequently, health care professionals are not fully informed.

In the second pattern, critical patients had a constructive relationship with their nephrologist with whom they felt comfortable discussing medication issues. These patients had a strong desire to have control over treatment decisions and, for instance, studied information leaflets, searched for information on the Internet, experimented with the dosage of the medication, or demanded alternatives with fewer side effects. They were also assertive with their nephrologist: “*I want to know what pills I am taking, and for what, and if they don’t work, I sound the alarm, because I do not want to swallow anything I do not need to.*” (participant 18). Another participant said, “*I don’t think I am a guinea pig, just take medication and wait and see if it works*” (participant 23). Barring some exceptions, nephrologists were open to participants’ suggestions.

These patients sometimes pointed out mistakes that they thought the nephrologists had made but did not dismiss their competence completely. One participant, for instance, disagreed about the best moment to take her antihypertensives. In her experience, her blood pressure was better controlled if she took her medication before dialysis, but her nephrologist insisted she took it afterward. A Dutch-Surinamese patient who did talk a lot with her nephrologist could nevertheless not help having the impression that Surinamese nephrologists were more engaged than their Dutch counterparts. The few participants who actively contemplated rejecting treatment and embraced the consequence of possibly dying (earlier) usually shared this open communication with the health practitioners. Catherine (Box 2), for example, has this type of relationship with her nephrologist.

In the third pattern, a disapproving patient seriously questioned or downrightly rejected the physician’s expertise. Several participants had had bad experiences with nephrologists (in training) who rigorously corrected abnormal laboratory results, sometimes completely turning participant’s treatment regimens on their head, although the participants were happy with their current treatment. Other participants felt not taken seriously or misunderstood: “From time to time, I think ‘*swallow this yourself just once, so you know how it feels’ because sometimes it is so difficult to explain.*” (participant 1). Several participants were furious with their nephrologist’s warnings about the risk of addiction to sleeping tablets. In their view, their nephrologists did not have their priorities right and completely disregarded the importance of a good night’s sleep or the nuisance of staying awake at night. Michael (Box 1), for example, has this type of interaction with his nephrologist.

#### Family and Others

Medication was not just a matter between patients and their health care professionals but also involved participants’ social environments. Partners or (grand)children helped participants with their medication. They reminded their loved ones to take their medication, helped to organize medication, picked up medication from the pharmacy, and stored spare medication in case of a visit from them. The involvement of family in participants’ medication was not always welcome. One Surinamese woman said that her niece advised her to use medicinal leaves as treatment, although she had stated on multiple occasions she preferred the medication prescribed by her nephrologist. Others deliberately avoided discussing their health with their family or other patients.

## Discussion

In this qualitative study, we gained insight into dialysis patients’ experiences with polypharmacy. Participants generally perceived medication as unpleasant, experienced many side effects, and, on top of that, often did not experience any positive effects of medication. However, they did accept its importance in preserving their health.

Although each theme is presented independently, it is impossible to ignore how they are intertwined and form coherent narratives in the context of participants’ life history, as is illustrated in the life histories of Michael and Catherine (Boxes 1 and 2). Michael, a man with a strong faith in God and traditional medicine, is very skeptical about his nephrologists and western medicine. His skepticism regularly causes friction in the relationship with his nephrologists as he ignores their advice and refuses to take his medication due to a perceived lack of health benefits. Instead, he treats himself with medicinal leaves from Suriname. In contrast, Catherine, a former teacher, believes in scientific knowledge and perceives her medication positively. She possesses a strong sense of autonomy and critically appraises her nephrologists’ suggestions. She has enough self-confidence to discuss her treatment openly with her nephrologists and, unlike Michael, is receptive to their suggestions.

Box 2 **Portrait of Catherine, an Elderly Dutch Woman Who Has Great Faith in Western Medicine. She is Open to the Suggestions of Her Nephrologists, but can Also be Very Critical of Them.**

Catherine was in her early 80s when we interviewed her. She lives on her own in a comfortable apartment in Amsterdam. She had so much to tell; she easily filled 2 long interviews. She speaks with the intonation of an actress, sometimes lowering her voice for emphasis, sometimes bursting out into roars of laughter, and giving hand-blown kisses to express appreciation of certain persons.

She married in her early 20s, had 3 children in 5 years, and divorced young because she felt constrained by a life revolving around motherhood only. When her children had grown up and left the parental home, she seized the opportunity and took on a job in sub-Saharan Africa, where she eventually stayed for 10 years, active in the education of adult women. Upon her return to the Netherlands, she continued to work with adult women until after retirement, but now works with migrants. She also obtained a university degree in cultural anthropology to provide her with a theoretical foundation for her work. She was brought up a Christian, but lost her faith when her father died in a traffic accident: ‘*I have beraten our Good Lord out of Heaven.*’

Her kidney disease only came to light in her late 70s when she suffered from fatigue. However, in hindsight, the first symptoms had appeared 6 year earlier. She called kidneys ‘*deceitful organs [… because] they do not hurt, and one does not feel ill, [the disease] sneaks up on you*.’

During the interviews, more than once she confessed weighing the burden of dialysis and polypharmacy against the benefits to her health. However, her children and also her grandchildren, who coax her into telling stories ‘about Africa’, and her lust for life in general are strong reasons to bear the discomforts of her treatment. She has opted for automated peritoneal dialysis (APD) treatment because she can receive this treatment at home at night, and it allows her maximal independence. She has stored her medication and bags with peritoneal dialysis (PD) fluid away in one room (with the door closed), and a nice piece of cloth is draped over the dialysis machine to hide it from sight. She receives support from her family. One of her grandsons had given her a box to keep her pills. A daughter learned to operate the dialysis machine, so they could go on holiday together.

She often discusses medication with her physicians and has a decisive opinion about whom she finds good and who fall short. Because of her high expectations of quality of life, she felt disappointed that the surgeon who inserted her PD catheter painted an overly optimistic picture of how much better she would feel after treatment. She had a major conflict with another physician who was worried she would become addicted to sleeping pills: “*I say, Fine! Nicely addicted. But let me sleep! … and then the doctor decides if my life is a lot more comfortable. That is not possible, is it? It makes me so angry!”* She did praise other physicians and a nurse, all mentioned by name, for their enthusiasm about health improvements, clarity, realism in their updates on her health, and willingness to apologize for medical errors. She also complimented a doctor who respected her putative wish to stop treatment at some time in the future, and a nurse who rested a hand on her to calm her. Despite her critical attitude towards physicians and the whole treatment, in the end, she is very co-operative and grateful for the medical care. What bothers her most is the loss of autonomy.

Our results corroborate the results from our quantitative analysis that polypharmacy negatively affects dialysis patients’ quality of life and perception of their health.^4^^,^^5^ However, this qualitative analysis allows an understanding of the underlying mechanisms. Patients must contend with different burdensome aspects of medication, such as logistical issues, side effects, and feelings of shame, dependency, and internalized resistance, which can amount to significant discomfort.^6^^,^^7^^,^^13^ On the other hand, patients might also underrate the positive effects of medication on their quality of life because most medications do not relieve symptoms and rather than reverse the process, only slow down the deterioration of their health.

The experiences with medication described by our participants largely mirror the experiences of kidney transplant recipients and other patients who are not receiving dialysis treatment with polypharmacy.^6^, ^7^, ^8^ Nevertheless, 2 important differences should be noted. First, they take the burden of polypharmacy relatively lightly because the burden of medication pales in comparison to the burden of dialysis. Similarly, the fear of having to endure the discomforts of dialysis can motivate patients with a kidney transplant to endure the discomforts of their immunosuppressive medication.^6^ Second, contrary to previous studies on patients’ experiences with medication, the participants in this study also emphasized the importance of medication to their health and the ways in which medication can alleviate symptoms, although they notice these positive effects only indirectly from laboratory results.^7^^,^^8^ Patients not only use these “external medicalized determinants of one’s health status” to appraise the efficacy of their medication but also to determine the efficacy of their dialysis treatment.^14^

The qualitative research gives better insight into how participants managed to mitigate the negative effects through effective coping mechanisms and integrate medication in their daily routines. The efforts to make medication and dialysis a “normal” part of life and to retain as much of “normality” form a recurrent theme in qualitative studies on dialysis patients.^12^, ^13^, ^14^, ^15^

The interaction with health care professionals formed part of these routines. Preferences for taking responsibility for treatment decisions varied considerably between participants. Some were actively involved in making decisions, while others accepted their nephrologists’ advice without questioning. A third group denied the nephrologists’ expertise. All relied on their nephrologists to interpret the measurements of health indicators to determine the effectiveness of medication. Other qualitative studies of patient–physician relationships only observed the first 2 patterns of interaction, but not the third where patients denied the physician’s expertise. If patients were found critical, it was ascribed to past negative experiences with health care professionals, for instance, because patients perceived a lack of empathy or because the health care professionals allegedly had financial interests in prescribing medications.^6^^,^^14^^,^^16^, ^17^, ^18^ For some patients, these negative experiences were a reason to avoid discussing medication with their clinicians, including medication problems and nonadherence, out of fear of being reprimanded or not being taken seriously.^6^^,^^17^^,^^18^ Although our participants also shared some negative experiences with inattentive physicians, their attitude toward physicians could partly be explained in holistic manner from their life histories, as suggested by Michael and Catherine (Boxes 1 and 2).

Our study identified several opportunities to improve dialysis patients’ experience with medication (Box 3). Nephrologists and patients together should evaluate the (negative) impact of medication on health and other aspects of patients’ lives continuously to make shared decisions about the most effective and least burdensome treatment. During discussions about medication with their patients, being aware that patients might have a different understanding of what ‘medication’ is can avoid misunderstandings. Pointing out the positive effects of medication that patients might not feel themselves can help to boost their motivation. Minor adjustments in the type of medication or route of administration can help to mitigate the impact of medication on quality of life or even improve it.

Box 3 **Practical Recommendations for Health Care Professionals to Manage Polypharmacy in Patients Receiving Dialysis Treatment.**

**Discussing medication with patients**

• Regularly discussing medication with patients at monthly intervals (eg, during out-patient visits or dialysis rounds) allows the timely identification of medication-related problems and may increase patients’ motivation.
• Evaluate not only the clinical effects of the medication but also its effects on daily life and the inconveniences it causes.
• Be aware that patients can construe different things as medication. This can cause miscommunication between patients and health care practitioners.
• Different interaction patterns between patients and health care professionals have possible consequences how patients discuss or deal with medication.
• Linking improvements in lab values or symptoms to medication can help to demonstrate the efficacy of therapies to patients and improve adherence (eg, diuresis has improved because of the increase in the dosage of diuretics). Repeatedly emphasizing the importance of medication/effects on health, even if the medication regimen is consistent, can increase patients' motivation to take their medication as prescribed.

**Managing medication regimens**

• Support patients in developing effective and acceptable medication routines.
• Allowing metabolic problems or symptoms more time to resolve by themselves rather than adjusting medication regimens immediately may avoid unnecessary changes to patients’ medication regimen.
• Adjusting patients’ dialysis regimens to resolve metabolic disturbances or reduce symptoms can be a good alternative to prescribing more medication.

**Reducing the negative impact of medication on quality of life**

• For patients with a fluid restrictions, prescribing medication that can be taken with little water or viscous food, like stewed apple, reduces the amount of fluid from the fluid ration patients have to spend on their medication.
• Limiting the number of times patients have to take medication, particularly during the day, and combining dialysis and medication routines reduces the time that patients have to think about their illness and spare them embarrassing moments of medication intake in the presence of others.
• There are several opportunities for medication to make patients’ life more comfortable (eg, to reduce symptoms, to facilitate more dietary liberties, or to make dialysis more comfortable) and capitalize upon them.

One strength of this study is its qualitative design. Participants were generally enthusiastic about the open questions that enabled them to share what is important to them, instead of a standard questionnaire. We believe that we established a good rapport with participants as they generally appreciated the time and attention received from the interviewers. Another strength is that our study population reflects the Dutch dialysis population in the distribution of age, gender, and home versus in-center dialysis. The biggest limitation is that qualitative research is, by definition, context dependent, and findings cannot be applied uncritically to other situations. Although polypharmacy is universal among dialysis patients, the experiences of our participants cannot be translated one-to-one to patients from different (cultural) backgrounds or different health care systems. For example, the financial burden of medication is relatively small for Dutch patients because almost all medication is reimbursed. Nevertheless, the challenges identified in the core themes are ubiquitous for most dialysis patients and therefore likely shared by patients in other settings.

In conclusion, polypharmacy negatively affected dialysis patients’ quality of life. These effects were, however, overshadowed by the burden of dialysis. Patients’ realization that medication is important to their health and effective coping strategies mitigated the negative impact of polypharmacy on quality of life. Future studies should aim to develop and evaluate strategies to improve dialysis patients’ experience of medication and shared-decision making between physicians and patients for effective (de)prescribing.

## References

[1] Alshamrani M., Almalki A., Qureshi M., Yusuf O., Ismail S. (2018). Polypharmacy and Mmedication-related problems in hemodialysis patients: a call for deprescribing. Pharmacy (Basel).

[2] Ghimire S., Castelino R.L., Lioufas N.M., Peterson G.M., Zaidi S.T. (2015). Nonadherence to medication therapy in haemodialysis patients: a systematic review. PLOS ONE.

[3] Manley H.J., Cannella C.A., Bailie G.R., St Peter W.L. (2005). Medication-related problems in ambulatory hemodialysis patients: a pooled analysis. Am J Kidney Dis.

[4] Colombijn J.M.T., Bonenkamp A.A., van Eck van der Sluijs A. (2021). Impact of polypharmacy on health-related quality of life in dialysis patients. Am J Nephrol.

[5] Chiu Y.W., Teitelbaum I., Misra M. (2009). Pill burden, adherence, hyperphosphatemia, and quality of life in maintenance dialysis patients. Clin J Am Soc Nephrol.

[6] Tong A., Howell M., Wong G. (2011). The perspectives of kidney transplant recipients on medicine taking: a systematic review of qualitative studies. Nephrol Dial Transplant.

[7] Mohammed M.A., Moles R.J., Chen T.F. (2016). Medication-related burden and patients’ lived experience with medicine: a systematic review and metasynthesis of qualitative studies. BMJ Open.

[8] Eriksen C.U., Kyriakidis S., Christensen L.D. (2020). Medication-related experiences of patients with polypharmacy: a systematic review of qualitative studies. BMJ Open.

[9] Tonelli M., Wiebe N., Manns B.J. (2018). Comparison of the complexity of patients seen by different medical subspecialists in a universal health care system. JAMA Netw Open.

[10] Fletcher B.R., Damery S., Aiyegbusi O.L. (2022). Symptom burden and health-related quality of life in chronic kidney disease: A global systematic review and meta-analysis. PLOS Med.

[11] van Eck van der Sluijs A., Bonenkamp A.A., Dekker F.W. (2019). Dutch nOcturnal and hoME dialysis Study to Improve Clinical Outcomes (DOMESTICO): rationale and design. BMC Nephrol.

[12] Tong A., Sainsbury P., Craig J. (2007). Consolidated criteria for reporting qualitative research (COREQ): a 32-item checklist for interviews and focus groups. Int J Qual Health Care.

[13] Guerra-Guerrerro V., Plazas P., Cameron B.L., Salas A.V., González C.G. (2014). Understanding the life experience of people on hemodialysis: adherence to treatment and quality of life. Nephrol Nurs J.

[14] Raj R., Brown B., Ahuja K., Frandsen M., Jose M. (2020). Enabling good outcomes in older adults on dialysis: a qualitative study. BMC Nephrol.

[15] Moreels T., Van de Velde D., Van Duyse S. (2023). The impact of in-centre haemodialysis treatment on the everyday life of older adults with end-stage kidney disease: a qualitative study. Clin Kidney J.

[16] Gilad L., Haviv Y.S., Cohen-Glickman I., Chinitz D., Cohen M.J. (2020). Chronic drug treatment among hemodialysis patients: a qualitative study of patients, nursing and medical staff attitudes and approaches. BMC Nephrol.

[17] Rifkin D.E., Laws M.B., Rao M. (2010). Medication adherence behavior and priorities among older adults with CKD: a semistructured interview study. Am J Kidney Dis.

[18] Ghimire S., Castelino R.L., Jose M.D., Zaidi S.T.R. (2017). Medication adherence perspectives in haemodialysis patients: a qualitative study. BMC Nephrol.

