# Supplementary material for: Polypharmacy and Quality of Life Among Dialysis Patients: A Qualitative Study
Source: Kidney Med. 2023 Nov 28;6(1):100749. doi: 10.1016/j.xkme.2023.100749 (PMC10777060; doi:10.1016/j.xkme.2023.100749)
Supplement: Supplementary File (PDF) — Table S1-S2 [file mmc1.docx]

**Supplementary materials**

**Table S1:** topic list of interviews

| In contrast to a survey with standard questions asked in a fixed order used in quantitative research, a qualitative research approach with open questions takes the form of a natural discussion with many follow-up questions. A degree of standardization is guaranteed by using a topic list; topics can be raised by the interviewer as they come up during the discussion, but, in the end, all topics must have been addressed. The topic list contained the following items:   - History of kidney replacement therapy, including dialysis in the context of full medical history (as told by patient) - Assessment of one’s health - Daily or weekly routines of medication use (on dialysis and non-dialysis days) / integration of medication in daily routines - Experience of medication (what effects are felt by participants) - Knowledge about medication and strategies to acquire knowledge - Interaction with healthcare workers (including around medication) - (Non)adherence - Changes in everyday life since the beginning of dialysis - Effect of dialysis on quality of life (and participants’ definition of ‘quality of life’) - Feeling of possible loss of agency - Feeling of possible loss of dignity - Positive and negative reactions from close and distant persons in social network - Possible feeling of inadequacy with close relatives and friends - Impact of Covid-19 on relative assessment of own health and relative exclusion from social life (Fear-of-missing-out) |
| --- |

**Table S2:** Original quotes in Dutch and translations into English

| **Resp** | **English translation** | **Original quote in Dutch** |
| --- | --- | --- |
|  | **Perception of medication** |  |
| 25 | *‘I feel so terribly dependent on it’* | *‘Ik voel me er verschrikkelijk afhankelijk van’* |
| 6 | *‘I have always learnt: you have to find your own solution.’* | ‘I*k heb altijd geleerd: je moet het zelf oplossen*’ |
| 1 | *‘I am part of nature but I swallow chemical garbage’* | *‘Ik ben natuur maar ik slik allemaal chemische rommel’* |
| 18 | *‘I would rather not take it [medication] [ …]. But I cannot live without it, so you can grumble all your like but that does not help’* | *‘Je neemt het liever niet […] Maar ja, ik kan niet zonder, dus je kunt wel sputteren, maar dat helpt ook niet.’* |
| 10 | *‘It’s part of daily life. I mean, there are also other people who have to take medication. It’s all part of the package.’* | *‘Het hoort bij het dagelijks leven! Ik bedoel je hebt ook andere mensen die medicatie moeten gebruiken… Dat hoort erbij’* |
| 12 | *‘It is my life-saver! I am so grateful that I […] still have this chance [in my life]. It is the medication …I’m for it’* | *‘Het is mijn levens verlenger! Ik ben zo dankbaar dat ik… Dat dit nog kan! Het is de medicatie… ik ga ervoor’* |
| 15 | *‘You have to take your medication because you would destroy yourself if you didn’t […] then you’ve had it.’* | *‘Je moet toch je medicijnen innemen, want je sloopt jezelf als je dat niet zou doen, […] dan ben je gewoon aan de beurt.’* |
| 3 | *‘For me "there is no other way. You must do this or you will have to put on a wooden coffin and your suffering will be over. But I’m not ready to do that.”* | *‘Ik heb zo van 'nou het is niet anders, het moet maar zo of je moet een houten kist aantrekken en dan heb je nergens meer last van'. Maar dat wil ik nog niet’* |
| 19 | *‘I just don’t like having to place those pills next to your plate everywhere [you go]. Maybe a sense of shame […] a kind of embarrassment that I happen to be the patient, the feeble one, the vulnerable one’* | *‘Ik vind het niet fijn gewoon dat je overal die tabletjes naast je bord moet leggen. Misschien wel een stuk schaamte’ […] een bepaalde gêne dat je jij dan net die zieke bent, die zwakke, die kwetsbare.* |
| 24 | ‘*I am not going to stand up in the middle of a [restaurant] to take pills, but I am not ashamed of it either. [Taking pills in public] doesn’t bother me at all’.* | *‘Ik ga niet midden in de zaal staan en pillen nemen. Maar ik schaam me er ook niet voor. Heb ik helemaal geen probleem mee.’* |
|  | **Medication routines and the impact of medication on daily life** |  |
| 27 | *‘You do not forget to eat, so, yes, this little basket is always on the table’* | *‘Je vergeet ook niet te eten. Dus ja, ik heb het mandje altijd op tafel staan.’* |
| 3 | *‘Sunday is pill day’* | *‘Zondag is pillendag’* |
| 16 | *‘It is automatic […] I don’t even think about it.’* | *‘Het gaat automatisch … ik denk er niet eens bij na.’* |
| 24 | *‘Everything in one go, gulp of water, done.’* | *‘Alles in een keer, slok water, klaar.’* |
| 19 | *‘Ideally, I have nothing to do with dialysis during the day. During the evening and at night, I am willing to have it, but during the day I just want to be free without having to think “Oh, I have to take a little pill”.’* | *Ik wil eigenlijk het liefste dat ik overdag helemaal niet met de dialyse bezig ben. Gewoon 's avonds en 's nachts wil ik het wel doen maar overdag wil ik gewoon mijn vrijheid en niet moeten denken van 'oh ik moet een tabletje slikken'* |
| 23 | *‘I take it [medication] and after that I forget I am ill’* | *‘Ik slik ze en daarna ik vergeet dat ik ziek ben’* |
|  | **Interaction with healthcare workers and social environment regarding medication** |  |
| 29 | *‘That doctor looks at which medication is good for me […] “Then that will be all right”, is what I say. Doctors know better than I do’* | *‘Die dokter gaat kijken wat voor medicijnen voor mij goed zijn. […]het zal wel goed zijn zeg ik. Doktoren weten het beter als ik.’* |
| 2 | *‘The doctor ordered me to’* | *‘Van de dokter krijg ik opdracht om’* |
| 1 | *‘From time to time I think “swallow this yourself just once, so you know how it feels”. Because sometimes it is so difficult to explain* | *‘Ik denk ook wel eens van slik het zelf eens een keer, dan weet je wat, hoe je dat ervaart. Want het is soms zo moeilijk uit te leggen’* |
| 18 | *‘I want to know what pills I am taking, and for what, and if they don’t work, I sound the alarm, because I do not want to swallow anything I do not need to’* | *‘Nou, ik wil weten wat ik slik en waarvoor ik het slik en als het niet helpt dan hang ik aan de bel, want ik wil niets slikken wat ik niet nodig heb.’* |
| 23 | *‘I don’t think I am a guinea-pig, just take medication and wait and see if it works’* | *‘Ik denk niet ik ben een proefkonijn van slik maar dat medicijn en we kijken of het werkt.’* |
|  | **Portrait Michael** |  |
| 4 | ‘*I am religious. I believe in God. […] I thought: you [the nephrologist] are not God. Only a doctor*’ | *Ik ben gelovig. Ik geloof in een god.[…] Ik dacht, jij [de nefroloog] bent geen god, maar een arts.* |
| 4 | *‘I thought, they [nephrologists] are just tinkering. They’re only human. They make plenty of blunders. I am glad they keep me alive and should be grateful for that. If it were not for the dialysis, I would be gone by now. [But] I don’t understand why they don’t look at the other side. Herbs and spirituality etc.’* | *‘Ik dacht toen van ze [nefrologen] proberen maar wat. Het zijn ook mensen. Ze blunderen ook veel hoor. Ik ben blij dat ze me in leven houden, ik moet er dankbaar voor zijn. Als het er niet was, dan was ik al weg. Ik snap niet dat ze niet naar de andere kant kijken, kruiden en voelen, etc.’* |
| 4 | ‘*Pills have not done me any good. […]* *I have tried all kinds of pills but it is just chemical garbage. As a patient you are desperate. They prescribe you pills like a guinea-pig. It felt as if the medication runs riot with my body*’ | *‘Pillen hebben niks goed gebracht. […] Ik heb alle soorten pillen geprobeerd, het is chemische troep. Je bent radeloos als patiënt, dan krijg je pillen, als proefkonijn, als of m’n lichaam ging haperen soms, door de pillen’* |
| 4 | *‘I am not going to follow it [the treatment]. I’ll go [to the Hereafter] only once, and when I go, I shalll go in peace. It is out of our hands’* | *‘Ik doe het toch niet. Ik ga maar een keer, en als ik ga dan ga ik in vrede. We hebben niets in de hand.’* |
|  | **Portrait Catherine** |  |
| 26 | *‘I have berated the Good Lord in heaven*’ | *‘Ik heb Onze Lieve Heer uit de hemel gescholden’* |
| 26 | ‘*Deceitful organs [… because] they do not hurt, and you don’t feel ill, [the disease] sneaks up on you*’. | *‘Achterbakse organen […] ze doen je geen pijn, je bent er niet ziek of beroerd van, het sluipt erin’* |
| 26 | *‘I say: Fine! I might be addicted, but let me sleep! […] and then the doctor decides if my life is a bit more comfortable. That is not possible, is it? It makes me so angry.’* | *Ik zeg: Prima. Lekker verslaafd. Maar laat me wel slapen! […] en dan bepaalt die arts of mijn leven wel of niet een stuk aangenamer is. Dat kan toch niet? […] Daar word ik zo boos van!* |
